# 

**Published:** 1891-05-16

**Authors:** 


### NOTICE TO CORRESPONDENTS.

All MS., Letters, Books for Review, and other matters intended for the Editor should be addressed The Editor, The Lodge, Porchester Square, London, W.
The Editor cannot undertake to return rejected M.S., even when accompanied by stamped directed envelope.

**Notice to Advertisers and Subscribers.**

All Advertisements, Orders for Copies of The Hospital, and Business Communications should be addressed to The Publisher, *not to the Editor*, The Hospital, 140, Strand, W.C.
The Cost of Subscription, post free, is as follows:—Per week, 1½d.; per quarter, 1s. 8d.; per half-year, 3s. 3d.; per annum, 6s. 6d. Foreign:—Per annum, 8s. 8d.; payable, in all cases, in advance.

**NOTICE.—The Index for Vol. IX. (ending March, 1891) is on sale at the Office of this Journal, price 2½d., post free.**

### General Charities.

[This Column is devoted to an account of work of charitable institutions other than Medical Charities. The Editor will be glad to receive reports or news of work at 140, Strand. *Philanthropy* should be plainly written on the envelope.]

Tickets for the festival service of the **CHURCH OF ENGLAND TEMPERANCE SOCIETY**, which is to be held at Westminster Abbey on Monday, the 27th inst., can be had on application to the Secretary, Mr. F. Eardley Wilmot, 9, Bridge Street, Westminster, S.W.

The Committee of the **BRITISH ORPHAN ASYLUM**, Slough, appeals for help in connection with the anniversary festival, which is to be held at the Whitehall Rooms, Hotel Métropole, on Thursday, May 14th, under the presidency of Mr. Alderman Davies. Offices, 62, Bishopsgate Street Within, E.C.

The Queen has expressed her satisfaction at hearing of the efforts which have been made to increase the efficacy of the branch of the **SOCIETY FOR PREVENTION OF CRUELTY TO ANIMALS** at Mentone in a very practical manner, by sending a donation in aid of its excellent and apparently much needed work. It is stated on good authority that during her stay at Grasse the Queen has seen with much pain the cruelty displayed towards animals in the district, and has suggested to the President of the branch of the Society at Cannes the advisability of sending an inspector to Grasse. No doubt the suggestion will at once be acted on.

The need of such an institution as the **EASTERN COUNTIES' ASYLUM FOR IDIOTS** is unhappily only too clearly shown by the fact that out of the 40,000 idiots said to be in England and Wales there are no fewer than 3,000 in the Eastern Counties. This Asylum, which was established, as long ago as 1859, for the care, education, and general training of idiots and imbeciles, whose power of brain has in so many cases been lost from their birth, had the good fortune to secure the advocacy of the Prince of Wales on its behalf at its recent festival dinner. He pointed out that notwithstanding the number of lunatic asylums which exist throughout the country, there are only five idiot asylums in the whole of England and Wales. The Eastern Counties' Asylum contains 220 beds, of which 200 are at present occupied. Like many other similar institutions its expenditure largely exceeds its certain income. A valuable addition, to the amount of £6,000, was made at the festival to its funds, and we understand that the management are appealing to the public to enable them to obtain an endowment fund of £50,000. No one certainly can be more deserving of help than those who from their birth have been so unhappily equipped for the battle of life.

In these days of extensive travelling, such an institution as the **RAILWAY OFFICERS AND SERVANTS' ASSOCIATION**, which has now been in existence for thirty years, should appeal to the hearts of every section of society. The enormous responsibility of those who are entrusted with the working of our train system is too widely known to need impressing on the public. It is often said that there is no safer place to be in than a passenger train; the saying is truer than many other excellent saws, and the truth of the assertion is shown by the fact that not more than one person, on the average, is killed in 10,000,000 journeys, or injured in more than 900,000. But if the general public meet with such encouraging immunity from danger, the servants of the various railway companies, from the very nature of their callings, cannot claim such exemption. The actual dangers which surround the lives of shunters and firemen are as numerous as they are varied, and in addition to the dangers to which they are constantly exposed, they too frequently fall victims to illnesses caused by the inclemency of our fickle climate. The objects of the Association are to give temporary, and in some cases permanent, assistance to railway servants in cases of accidents or incapacitating illness; to provide them with annuities in old age; and at their death to relieve the necessities of their families. Offices, 21, Finsbury Pavement, E.C.

The donations at the recent anniversary dinner of the **ROYAL LITERARY FUND** amounted to £1,100, including a fifty-fourth donation of £105 from the Queen. As Lord Halsbury, the Chairman, said: "The public generally owe to literary men a debt which is not sufficiently recognised, and can never be paid, seeing that literature embraces every department of human knowledge and imagination." The Lord Chancellor might we think, with all fairness, have twitted literary men themselves with having a want of practical sympathy for their fellow-workers. If we are not mistaken, the names of most of the successful novel writers—the one class of author which makes money nowadays—are conspicuous by their absence from the list of supporters of the fund. If authors who can afford to show more than an abstract sympathy for their fellow-workers fail to do more, it is hardly astonishing, however regrettable the fact may be, if the general public are contented with a mere abstract admiration of that to which they are so indebted. Mr. Justice Jeune and Sir Edwin Arnold both echoed a sentiment which is undoubtedly beginning to awaken in the minds of some, that in the widened diffusion of literature, which is hailed by many as one of the glories of the age, there is a danger, none the less real because only visible to thoughtful people, of degeneration in earnestness of purpose and in grace of style.

### Scraps and Gleanings.

Dr. Muriel Maitland King has originated a new cure for corpulence.

The Duke of Rutland has given £300 towards the fund for re-building the Derbyshire Infirmary.

The Irish Distressed Ladies' Fund, 17, North Audley Street, has received a donation of £25 from the Clothworkers' Company.

At a meeting of the Urban Sanitary Authority of Stockton, it was resolved to erect a fever hospital to contain 32 beds at a cost of £7,300.

Dr. Theodore Thomson, Medical Officer of Health for Sheffield, has been appointed to an inspectorship in the Medical Department of the Local Government Board.

Public meetings are being held in many of the towns near Derby for the purpose of discussing the question of raising funds for the re-building of the Derbyshire Infirmary.

At a meeting of the Association of Public Sanitary Inspectors of Great Britain on Saturday, a paper was read by Dr. B. W. Richardson, the President, on "National Main Drainage."

The Hospital of St. John and St. Elizabeth for Incurables reports 50 beds fully occupied. One hundred new cases were received last year, 65 were discharged, and 35 died. The receipts were £1,981.

The festival dinner of the West Ham Hospital was held at the Albion Hotel, Aldersgate Street, on the 22nd ult. Sir Thomas Fowell Buxton presided, and subscriptions amounting to £1,715 were announced.

The Countess of Shaftesbury has, with the consent of her son, presented the lease of the Belfast Royal Hospital to the Corporation for "ten thousand years" at the nominal rent of 2s. 6d. a-year.

The Mayor of Sheffield has opened a fund to meet the necessities of a great number of the inhabitants who are suffering from influenza, and are unable to provide the remedies to enable them to regain their health.

During the month on April, 3,887 patients were relieved at the Great Northern Central Hospital, of which 1,489 were new cases and 281 accidents, 30 in and 21 out-patients could not be admitted for want of room.

On May 2nd a performance of "Still Waters Run Deep" was given in the Royal Park Hall, Camden Town, in aid of the funds of University College Hospital. The performance was under the direction of Mr. T. T. Lentner.

Among the contributors towards the fund to be collected at the festival dinner in aid of the North-Eastern Hospital for Children are Mr. T. G. Barclay, £200; Mr. A. Rüffer, of Lombard Street, £100; and Mr. M. Rüffer, £50.

The report for 1890 of the North Cambs. Cottage Hospital shows that 163 persons participated in the benefits of the Hospital. The income of the Hospital was £1,165 2s. 2d., and the expenditure £1,068 12s., leaving a balance in favour of the Hospital of £96 10s. 2d.

Miss Annette Benson, M.B., B.Sc.Lond., has been appointed resident Clinical Assistant at the Paddington Infirmary. Miss Benson was a student at the London School of Medicine for Women, towards the fund for altering and enlarging which the Clothworkers' Company have just given £50.

A meeting was held at Chester Town Hall for the purpose of considering the propriety of establishing a memorial to the late Colonel Humberston. A legacy of £500 had been left by the colonel to the Infirmary, and it was resolved that the legacy should form the nucleus of a fund to be devoted to the erection of a new wing to the institution.

The presentation of medals and certificates awarded by the Duchess of Westminster to candidates having successfully passed the examinations of the National Health Society on "Domestic and Personal Hygiene," "First Aid to the Injured," "Home Nursing," and "Elementary Physiology," took place on Saturday in the Reubens' Gallery, Grosvenor House.

The annual report of the Samaritan Hospital for Women, Nottingham, states that the work done during 1890 was of a most satisfactory character, 136 in-patients and 2,124 out-patients having been treated. The year began with an adverse balance of £318 0s. 9d., and the year has closed with a deficit of £551 17s. 10d. There has been a falling off in donations to the hospital.

A meeting was held in Leeds on the 1st inst. for the purpose of appealing to the inhabitants of Leeds on behalf of the Royal Bath Hospital, Harrogate. A large percentage of the patients came from Leeds, while the contributions from Leeds were very small. There is a debt on the building of £11,397, the interest on which entails an annual charge of £450, about one-fifth of the income of the hospital.

The long foggy winter has caused great mortality among the animals at the Zoo. The weather, however, was not accountable for the death of the African black rhinoceros, as the post-mortem examination made by Mr. Beddard showed that not only was it suffering from cancer in the stomach, but also from heart disease, which was the actual cause of death. This animal had lived in the gardens for nearly 23 years.

Before closing their final session in Washington, the American Medical Association officially expressed the opinion that Koch's lymph was a valuable remedy for consumption. Dr. Von Ruck showed that at his sanatorium at Nashville the lymph had done excellent work, and several patients were cured by it. The American Medical Association wants Congress to create a Cabinet officer with the title of Secretary of Public Health.

The Poplar Hospital Benevolent Association has been investigating charges which have been made with reference to the treatment of in-patients at the Poplar Hospital. The statements of about a dozen former in-patients have been taken, in all of which complaints are made about the quality and quantity of the food supplied. It is intended to send a copy of the statements to the Charity Commissioners with a view to an inquiry being held.

Dr. Bernard M. Pahl a noted Prussian physician, who committed suicide at Findlay, Ontario, endeavoured to benefit his profession by recording the varying effects of the poison which he used. To secure his end, about three o'clock one morning he took fifty-three morphine pills, and then lay down with his face to the wall, upon which he scribbled the results with a pencil. He calculated that death would ensue in seven hours, but he expired at half-past eight.

### The Student of Medicine.

[All communications for this Supplement should be addressed to The Editor of The Hospital, at 140, Strand, with the words, "Students' Column," written in the left hand top corner of the envelope.]

### LEADING ATHLETES.

Mr. Percy Fernivall, the world-renowned bicyclist, was born in London in the year 1867. He was educated at University College School, subsequently having registered as a medical student he entered St. Bartholomew's Hospital in 1885. He learnt to ride a bicycle in 1879 and started racing in 1882, when he won his school two mile bicycle handicap by over a quarter of a mile. In the following year he won the first open bicycle handicap at Cambridge, riding down from London in the morning, winning the four mile race in the afternoon, and riding back to town in the evening. In 1885 he rode a racing bicycle for the first time. On June the 15th of this year, at the Cambridge University Bicycle Club meeting in the two miles invitation scratch race, he beat the "flying" mile bicycle record, time, 2 min. 41 2-5 sec., and with Mr. G. Gatehouse on a tandem tricycle beat every record for that class of machine up to five miles. The times were as follows: A quarter of a mile in 41 sec.; half a mile in 1 min. 21 sec.; three-quarters of a mile in 2 min. 4 sec.; one mile in 2 min. 47 1-5 sec.; two miles in 5 min. 48 sec.; three miles in 8 min. 39 sec.; four miles in 11 min. 30 3-5 sec., and five miles in 14 min. 22 3-5 sec. On the 11th of July he won the mile tricycle championship in 2 min. 58 1-5 sec., thus beating the tricycle record. After this he went to America with a team of English cyclists, and on the 9th of September at the Springfield Meet he won the one mile bicycle race, the so-called "Amateur Championship of the World;" and at the same meeting he beat all the tricycle records from one to five miles, the times being as follows: One mile in 2 min. 58 2-5 sec.; two miles in 6 min. 3 4-5 sec.; three miles in 9 min. 8 3-5 sec; four miles in 12 min. 15 1-5 sec., and five miles in 15 min. 18 3-5 sec. In 1886, on April 26th, at Norwich, he beat the quarter of a mile bicycle grass record in 39 3-5 sec. On May 22nd, at the Alexandra Palace, he won the Five Miles International Challenge Shield, on June 14th the one mile tricycle championship, on June 26th the one mile bicycle championship, and on July 24th the five miles bicycle championship. In the same year, on August 23rd, at Long Eaton, he beat the quarter, half, three-quarters, and one mile bicycle records; his times were 37 2-5, 1.16, 1.53 4-5, and 2.32 2-5. On September 18th, at the Kennington Oval, he carried off the Surrey B.C. Challenge Cup ten

### THE STUDENT OF MEDICINE—(*continued*).

miles bicycle grass race, and beat the record; time, 33 min. 40 2-5 sec.; also on the same day he won the quarter of a mile, making the mile record on grass in 39 sec. In the following year, 1887, on September 10th, he won the Surrey B.C. Challenge Cup (ten miles) for the third time in succession, beating the ten miles bicycle grass record in 32 min. and 36 4-5 sec. On September 14th, at the Crystal Palace, he beat the starting quarter-mile bicycle record; time, 37 1-5 sec. On September 22nd, at Surbiton, he beat the bicycle records from 11 to 25 miles; times, 11 miles in 32.7 3-5, 15 miles 43.59 2-5, 20 miles, 58.50 2-5, and 25 miles in 1 hr. 13 min. 49 3-5 sec. Although owing to the great improvement in the make of machines these records of a few years back have now been beaten, Mr. Furnivall's times for the 23, 24, and 25 miles have not as yet been surpassed.

### APPOINTMENTS.

At the Middlesex Hospital, Archibald Kidd, M.R.C.S., L.R.C.P., has been appointed house surgeon. John Panting, M.A. Camb., M.R.C.S., L.R.C.P., house physician. At the North-West London Hospital, Kentish Town Road, C. H. Fowler, M.R.C.S., L.R.C.P., has been appointed resident medical officer, and W. H. Vickery, M.R.C.S., L.R.C.P., assistant medical officer. At Guy's Hospital Dental School, A. D. Fripp, M.B., B.S., and H. F. Lancaster, M.D. Brux., M.R.C.S., L.R.C.P., have been appointed anæsthetists. At the Bridgewater Infirmary, T. D. H. Holmes, M.B., C.M. Edin., has been appointed house surgeon. At the Devonshire Hospital, Buxton, E. Valentine Gibson, M.B., C.M., has been appointed house surgeon. At the General Infirmary, Leeds, A. G. Hebblethwaite, M.R.C.S., L.R.C.P., has been appointed house physician. At the Norfolk and Norwich Eye Infirmary, C. J. Muriel, L.R.C.P. Lond., M.R.C.S., has been appointed assistant surgeon. At the Manchester Workhouse, W. J. Potts, L.R.C.P., M.R.C.S., has been appointed resident medical officer. At the Queen's Hospital, Birmingham, Leonard Parker Gamgee, M.R.C.S., L.R.C.P., has been appointed house surgeon. At the Royal Asylum, Edinburgh, J. N. Eustace, M.B., C.M. Dub., has been appointed clinical assistant. At the Swansea Hospital, T. Campbell Grey, M.R.C.S., L.R.C.P., has been appointed resident medical officer. At the St. George's and St. James' Dispensary, H. W. Dumergue, M.A., M.D. Cantab, M.R.C.S., has been appointed third honorary physician. At the St. Marylebone General Dispensary, 77, Welbeck Street, W. J. Radford, L.R.C.P., M.R.C.S., has been appointed resident medical officer. At the Tuam Workhouse, John E. Dowling, M.D.Irel., has been appointed medical officer. Dr. Peter Horrocks, of Guys, F.R.C.P., and Examiner in Obstetrics at the Royal College of Surgeons, has been unanimously elected Physician for the Eastern Division of London in the Royal Maternity Charity. At the North-West London Hospital, C. H. Fowler, M.R.C.S., L.R.C.P., has been appointed resident medical officer, and W. H. Vickery, M.R.C.S., L.R.C.P., assist. resident med. officer.

### VACANCIES.

The following vacancies are announced this week, the amount of salary in each case being given after the name of the institution:

**Resident Medical Officers.**

Borough of Plymouth. Medical Officer of the Borough Lunatic Asylum—Salary £400, &c.
Birmingham City Asylum, Resident Clinical Assistant.
Birmingham City Asylum, Assistant Medical Officer—Salary £120, &c.
Cumberland Infirmary, Carlisle, House Surgeon—Salary £70, &c.
Great Yarmouth Hospital, Resident House Surgeon—Salary £90, &c.
Hants County Asylum, Knowle, Fareham, Third Assistant Medical Officer—Salary £100, &c.
Holloway Sanatorium Hospital for the Insane, Virginia Water, Fourth Assistant Medical Officer.
Parish of St. Leonard's, Shoreditch, Resident Assistant Medical Officer for the Workhouse and Infirmary—Salary £100, &c.
Royal Westminster Ophthalmic Hospital, King William Street, West Strand, House Surgeon.
Torbay Hospital and Provident Dispensary, Torbay, Junior House Surgeon and Dispenser.
Warneford Hospital, Leamington Spa, House Surgeon—Salary £100, &c.

**Non-Resident Medical Officers.**

Boyle Union (Gurteen Dispensary), Medical Officer—£110, and fees.
Cancer Hospital (Free), Fulham Road, S.W., Honorary Pathologist; also a Surgeon.
Derby Amalgamated Friendly Societies' Medical Association, Assistant Surgeon—Salary £160, with additional fees.

### THE STUDENT OF MEDICINE—(*continued*).

Eccles and District Medical Association, Assistant Medical Officer.
Glamorganshire and Monmouthshire Infirmary and Dispensary, Cardiff, Honorary Pathologist; Honorary Dental Surgeon.
Queen's College, Birmingham, Professor of Medicine.
Salisbury Infirmary, Dispenser—Salary £105.

### PASS LISTS.

#### Society of Apothecaries of London.

The following passed the first examination in Chemistry, Materia Medica, Botany, and Pharmacy: R. Hall, University of Durham.

The following passed in the subjects indicated:—

Materia Medica, Botany, and Pharmacy.—H. K. C. Delamotte, Edinburgh; T. Hopps, Manchester, Owens College; J. Edwards, London Hospital; G. H. Nowell, B.A., Cambridge and Westminster Hospital.

Materia Medica and Botany.—F. K. Rouse, University College.

The following passed the second examination in Anatomy and Physiology: D. Berne, Sydney University; E. P. Hewitt and J. Garrett, St. Mary's Hospital; P. H. Best, B.A., Cambridge and University College; F. Kennedy, Edinburgh University; B. H. Carpenter and W. J. Woods, St. Bartholomew's Hospital; C. A. Marrett and L. C. L. Raymond, Charing Cross Hospital; H. J. Neatby, Leeds, Yorks College; C. D. Garrett and G. H. Nowell, B.A., Westminster Hospital; E. L. Greene, Royal Free Hospital; H. Herbert, London Hospital; R. M. Wright, Sheffield.

Anatomy.—G. C. Schultz, St. Mary's Hospital; J. F. W. Waters, Middlesex Hospital; H. C. Venis, Calcutta and St. Mary's Hospital.

Physiology.—G. E. Douglas and S. Langton, St. Mary's Hospital; W. R. Fisher and J. Joyle, London Hospital; G. Lowsley, St. Bartholomew's Hospital; J. M. Gleeson, Dublin and London Hospital; T. W. O'Reilly, St. Thomas's Hospital; C. C. Pratt, St. George's Hospital.

The following candidates passed in Surgery: W. Anderson, M.D., California, San Francisco; W. H. Kershaw, F. J. A. Baldwin, and R. J. Colmer, London Hospital; G. F. Knipe, Liverpool University College; L. J. Minter, King's College; J. F. Brown, B.A., M.D., C.M., Toronto; W. H. Savery, Sheffield; H. B. Falconar, King's College; H. N. A. Taylor, B.A., Cambridge University and Norfolk Hospital; A. F. Gervis, St. Thomas's Hospital; C. F. Warren, St. Mary's Hospital; M. J. Houghton and E. C. Wimberley, Birmingham, Queen's College.

The following passed in Medicine, Forensic Medicine, and Midwifery:—C. D. Holmes, Liverpool University College; H. de V. Stacpoole, St. Mary's Hospital; H. Knevitt and F. C. Wood, L.S.A., London Hospital; G. R. Stilwell, St. Thomas's Hospital; A. W. Read, St. George's Hospital; H. N. A. Taylor, B.A., Cambridge University and Norfolk Hospital; A. Richardson, Edinburgh University; W. A. Williams, L.M.S., Calcutta and Middlesex Hospital; E. M. Rooke, Guy's Hospital; P. Sharp, King's College.

The following passed in the subjects indicated:—

Medicine and Forensic Medicine.—R. S. Freeland, L.R.C.P., M.R.C.S., Guy's Hospital; G. F. Knipe, Liverpool University College.

Medicine and Midwifery.—R. Jackson, London Hospital; B. F. Parish, St. Mary's Hospital; C. P. Morgan, Guy's Hospital; H. J. Thomas, Bristol Medical School and Guy's Hospital.

Forensic Medicine.—E. A. Humphreys, Manchester, Owen's College.

Midwifery.—S. H. R. De Groot, King's College; A. G. Keeling, St. Thomas's Hospital.

Messrs. Colmer, Holmes, Houghton, Knevitt, Minter, Sharp, Stacpoole, Stilwell, Taylor, Wimberley, and Wood were granted the diploma of the society qualifying for registration and entitling them to practice surgery, medicine, and midwifery.

#### Royal Colleges of Physicians and Surgeons of Edinburgh and Faculty of Physicians and Surgeons of Glasgow.

The following shows the result of the April examinations held in Glasgow:—

First Examination.—Of 41 candidates the following 29 passed: Kate K. Paton, Glasgow; Robert P. Snodgrass, Montreal; Stanley H. Oulton, Liverpool; Janet R. Wells, Glasgow; Catherine Howie, Glasgow; Wm. E. Blakeley, Aughnacloy; John M'Lean, Glasgow; Malcolm Stewart, Kilwinning; Henry Marmion, County Down; Wm. Bond, Lancashire; Thomas B. Whitelaw, Kirkintilloch; Herbert P. Weston, Manchester; Gertrude N. O'Flaherty, Galway; Abraham J. Boulger, Colchester; Jean M. Grant, Banff; James Fenwick, Accrington; John D. Ballantyne, Dundee; Thomas J. Evans, New Quay, Wales; Thomas Heaps, Preston; Thomas French, County Cork; Richard C. Wilson, Birkenhead; Martin P. Corkery, Bombay; William Corkey, County Armagh; Luther T. Myers, Yorkshire; Cuthbert Rutherford, Northumberland; James H. Robinson, Southport; Samuel T. Brooks, Farnworth; Stanley B. Siddall, Stanley, Liverpool; and George Hill, Glasgow.

Second Examination.—Of 50 candidates the following 30 passed: James P. T. Burke, Ireland; Henry de Courcy O'Neale, Barbadoes; Alexander M'Afee, B.A., Ballymoney; Henry Joseph Cosgrove, Dumfries; William M. Fox, Cork; Robert Martin, County Down; Andrew M. Stewart, Alloa; Andrew J. M'Mickle, County Tyrone; John M. M'Millan, Argyllshire; George M. Wilcockson, Derbyshire; Thomas W. Waddell, Demerara; Janette F. A. Wallace, Inverness-shire; Thomas Dawson, Mid-Lothian; George C. Giles, York; Samuel W. Pitcher, Talbot, Australia; John Lamont, Argyllshire; Reginald H. Wright, Cheshire; Henry L. M'Culloch, Melbourne; William L. Crewdson, Edinburgh; Rees T. Jones, Glamorganshire; John C. Edwards, Denbighshire; Ralph T. Clark, Harrington; William Walker, Aberdeenshire; Jeanie Grant R. Duggan, Edinburgh; John Featherstone, Northumberland; Archibald M'Kellar, Glasgow; Michael Laby, Limerick;

### THE STUDENT OF MEDICINE—(*continued*).

Thomas C. Hunter, Glamorganshire; Arthur James Neill, Melbourne; and D. C. Lionel Williams, Breconshire.

Final Examination.—Of 36 candidates the following 15 passed: Daniel Doherty, Buncrana, Derry; Thos. Marshall, South Shields; Edith Mary Brown, Croydon; John Lane Gurrane, Fermoy; George Billing, Blackpool; Henry Collier, Carbrook; John Hodgson, Burnley; Edmund Hartley, Heywood; Jogendra nath Banerjee, Bengal; Charles N. Macquarie, Kilmuir; C. Blair Lucas, Carmyle; Henry D'Arnim Blumberg, Edinburgh; Joseph E. Joner Pegg, Birmingham; P. Algernon Shore, Walsall; and Simon Martyn, Bombay.

#### Edinburgh University.

The following is the official list of candidates who have passed the first professional examination in March: W. D. Adams, C. C. Aitken, H. de M. Alexander, James Anderson, R. W. Beesley, William Begg, B.A., William Bethune, J. L. Bevans, J. E. Bowes (with distinction), F. W. Broadbent, R. T. Bruce, Morton Burnet, A. P. Chapman, Joseph Cookson, B. R. Craig-Christie, Edward Crarer, J. M. Dalziel, J. R. Dodds, H. C. Dougall, H. W. Dun, Alexander Edwards, H. P. Elliot, David Evans, Thomas Finlay, John Forbes, J. V. Forrest, J. S. Fraser, Simon Fraser, W. H. Gaunt, Thomas Gibson (Australia), J. D. F. Gilchrist, M.A., P. E. H. Giuseppi, J. H. Glover, J. E. Good, D. J. Graham, J. H. K. Griffiths, William Haig, Henry Halton, Cooper Hardcastle, John Harris, R. T. Herdman, Arthur Heys, Edward Higinbotham, C. W. Holme, Archer Hosking, John Hume, F. C. M. Hutchinson, T. H. Jameson, Henry Jones, T. H. Jones, J. C. Kay, J. W. Keighley, B. C. Kelly, T. B. Kenny, J. W. Kippen, D. F. Laidlaw, John Laurie, T. G. Lewis, G. R. Livingston, T. G. Lusk, W. C. W. M'Dowall, C. J. R. MacFadden, John Maciver, J. C. Mackenzie, W. H. Mackenzie, Roderick Mackinnon, J. G. Macmillan, Donald Matheson, Sidney Messulam, Joseph Michael, C. H. D. Moore, R. S. Mowat, M. G. Naidu, B.A., F. B. Oliphant, David Orr, E. L. Owen, J. L. Owen, G. W. S. Paterson, C. W. Peach, Fred. Porter, W. A. Potts, B.A.; F. G. Proudfoot, J. C. Rait, David Rankine, M.A., A. M. Rattray, William Riach, R. G. Robson, W. C. Rowlands, Robert Samut, James Scott, R. G. Selby, J. H. Seon, T. D. S. Shaw, T. R. S. Sibbald, S. S. Skinner, B.A., George Smith, M.A., S. H. Smith, H. W. Somerville, W. L. Stevenson, Thomas Stuart, J. W. Sutcliffe, F. W. Taylor, A. G. P. Thomson, A. S. Trapaga, D. A. Turkhud, W. E. Turnbull, G. D. de Waal, James Watson, W. H. Watson, Daniel Watters, E. C. Watts, J. R. Whait, Alexander Whytt, P. W. Wilkinson, E. D. Williams, J. M. Wishart, J. B. Wood, J. J. H. Wood, Z. M. Zorab.

Second Professional Examination.—The following is the official list of candidates who have passed this month: G. W. Anorum, G. B. Anderson, W. C. Anderson, M.A., J. R. Armstrong, R. H. Armstrong, W. D. Barrow, Arthur Beecroft, H. P. D'A. Benson, C. H. Bond, M. R. Bow, D. H. Bure, George Butters, Albert Cameron, Daniel Campbell, W. S. Campbell, Featherston Cargill, J. F. Carruthers, J. G. Christie, M.A., P. G. Cilliers, G. P. Coldstream, H. D. Coles, A. B. J. Coope, James Cowie, J. W. Craig, S. G. Davidson, M.A., J. L. Dick, George Dickson, Alex. Douglas, M.A., R. W. Duncan, G. H. Dupont, D. C. Edington (with distinction), C. R. Edmondson, J. J. Evans, J. H. Ewart, Arthur Fells (with distinction), J. L. Ferris, William Fitzgerald, W. T. Fox, F. W. Foxcroft, David Fraser, J. A. Fullarton, Archibald Gardner, T. G. Goldie-Scott, W. B. Gow, T. A. Granger, H. F. Green, Vincent Green, G. K. Grimmer (with distinction), W. B. Harry, J. B. Hawthorn, T. B. Hearder, D. B. Hewat, R. L. Guthrie, J. A. Hamilton, L. E. Hardy, F. J. Hare, B.Sc., George Hodges, G. H. Hogg, George Home (with distinction), Robert Hutchison (with distinction), F. H. G. Hutchinson, S. P. Hyam, J. H. Johnston, J. L. Jones, H. W. G. Lander, Thomas Lawson, Frederick Lishman, H. C. Lloyd, G. O. M. Lunt, James M'Clew, James M'Donald, James Maclean, M.A., A. H. M. Macmorran, G. W. F. Macnaughton (with distinction), P. C. Malabre (with distinction), J. C. Maxwell, M.A., Raymond Maxwell, David Melville, J. E. Moorhouse, M.A., B.Sc., D. G. M. Munro, J. H. Murray, P. W. Nicol, Athelstane Nobbs, A. A. O'Hara, I. J. H. Oldmeadow, John Owen, G. W. Park, Charles Parker, F. R. Patterson, H. J. Pechell, W. H. Pimblett, C. F. Ponder, Robert Proudfoot, M. M. Rattray, G. P. Richards, Alistair Robertson, H. A. Robertson, J. F. Robertson, William Robertson, J. L. Russell, W. A. Rutherford, William Scott, Thomas Sidebottom, A. T. Simpson, J. P. Somerville, A. C. Stamberg, St. John Stanwell, Riccardo Stephens, A. G. Talbot (with distinction), M. W. Talbot, G. D. S. Thom, J. L. Thompson, J. B. Thomson, A. C. Turner, C. C. B. Tyrie, A. H. H. Vizard, F. J. Walden, Andrew Walker, M.A., John Wallace, D. C. Watson, J. K. Watson, W. T. Wearing, Evan Williams, G. J. Williams, Reginald Williams, J. C. Wilson, Meredith Young, and William Young.

### EVENTS FOR THE MONTH.

#### Students' Medical Societies.

University College Medical Society.—May 27th, paper by H. Head, Esq., M.A., "Some Aspects of Referred Pain. Pathological—Degenerations of the Spinal Cord. Histological—Nerve Cells." Chairman, Professor E. A. Schafer, F.R.S.

Westminster Hospital Guthrie Society.—May 14th.

#### Cricket.

Guy's Hospital (First Eleven).—May 16, v. R.I.E. College, at Cooper's Hill; 23rd, v. Crystal Palace, at Crystal Palace; 27th, v. Christ's College, Finchley, at Finchley; 30th, v. Bickley Park, at Bickley. Second Eleven.—May 16th, v. King's College Hospital, at King's College; 20th, v. Dulwich College, at College; 23rd, v. Forest School, at School; 30th, v. Wimbledon School, at School.

London Hospital.—May 16th, v. Brentwood, at Brentwood; 18th, v. Ealing, at Ealing; 20th, v. St. Anne's, at Virginia Water; 23rd, v. Surbiton, at Surbiton; 30th, v. Buckhurst Hill, at Buckhurst Hill.

